# Maternal satisfaction with reduced postnatal length of stay in Brussels: evidence from the KOZI&Home program

**DOI:** 10.1186/s12884-023-05740-0

**Published:** 2023-06-26

**Authors:** Amber Stas, Maria Breugelmans, Lynn Geerinck, An Spinnoy, Sven Van Laere, Leonardo Gucciardo, Monika Laubach, Gilles Faron, Katrien Beeckman

**Affiliations:** 1 Faculty of Medicine and Pharmacy, Vrije Universiteit Brussel, Brussels, Belgium; 2 Departement of Obstetrics and Prenatal Medicine, Universitair Ziekenhuis Brussel, Brussels, Belgium; 3 Maternity Ward UZ, Brussels, Belgium; 4 Interfaculty Center Data Processing & Statistics, Universitair Ziekenhuis Brussel, Brussels, Belgium; 5 Nursing and Midwifery, Centre for Research and Innovation in Care, Midwifery Research Education and Policymaking, Universiteit Antwerpen, Antwerp, Belgium; 6 Department of public health, Nursing and Midwifery Research Group, Universitair Ziekenhuis Brussel, Vrije Universiteit Brussel, Brussels, Belgium

**Keywords:** Early discharge, Integrated care, Length of stay (LOS), Postpartum Period, Patient satisfaction

## Abstract

**Background:**

Reducing the length of stay (LOS) after childbirth is a trend, including cost savings, a more family-centered approach and lower risk for nosocomial infection. Evaluating the impact of reduced LOS is important to improve the outcomes of care, which include maternal satisfaction. The aim of this study was to compare the maternal satisfaction, before and after the reduced LOS.

**Methods:**

This study was conducted in the University Hospital Brussels, before and after implementing the KOZI&Home program (intervention). This KOZI&Home program consisted of a reduced length of stay of at least one day for both vaginal delivery and caesarean section. It also included three extra antenatal visits with the midwife, preparing for discharge and postnatal home care by an independent midwife. Women completed a questionnaire, including the Maternity Satisfaction Questionnaire (MSQ) and Home Satisfaction Questionnaire (HSQ), respectively at discharge and two weeks postpartum. Satisfaction was split into five dimensions: ‘Midwives time investment’, ‘Provision of information’, ‘Physical environment’, ‘Privacy’ and ‘Readiness for discharge’. A combination of forward and backward model selection (both directions) was used for statistical analysis.

**Results:**

In total, 585 women were included in this study. 332 women in the non-intervention group and 253 women in the intervention group. Satisfaction with ‘provision of information’ at home had a higher mean score of 4.47/5 in the intervention group versus 4.08/5 in the non-intervention group (*p* < 0.001). Women in the KOZI&Home group were more satisfied regarding ‘privacy at home’ (mean 4.74/5 versus 4.48/5) (*p* < 0.001) and ‘readiness for discharge’ (*p* = 0.02).

**Conclusion:**

The intervention was associated with a higher score in some of dimensions of satisfaction. Our study concludes that this integrated care program is acceptable for postpartum women and associated with some favourable outcomes.

## Introduction

The postpartum period is the time after birth when the physiologic changes related to pregnancy return to the nonpregnant state, most often defined as six to eight weeks after given birth [1, 2]. The help and care during this stage is important for the mental and physical health of women and child, and should not be neglected [3, 4]. The most obvious change in postpartum care in almost all developed countries is a reduced length of stay (LOS) after childbirth [5]. In the 1950s, hospital stays of 11 to 14 days were not unusual, while currently average stays of three days or less are common in many western countries [6]. In Belgium the standard LOS after childbirth is four days [6, 7]. This is higher in comparison with other countries and higher than the average of the OECD (Organisation for Economic Co-operation and Development) countries, which has an average LOS of 3 days [6, 8, 9].

In addition to cost savings [10–12] the advantages of reducing LOS postpartum are well documented: a more family-centered approach to provision of care, lower risk for nosocomial infection, greater paternal involvement and less conflicting advice about breastfeeding [7, 13–15]. Disadvantages of reduced LOS include: problems establishing breastfeeding, late detection of disorders, and dissatisfaction of the mother [7, 14, 16–19].

Satisfaction with postpartum care was shown to be an important factor on parenting self-efficacy, breastfeeding and healthcare outcomes [20] and is considered to be an important indicator of healthcare quality and a factor in influencing treatment compliance and success [21]. Previous studies, conducted in well developed countries in Europe and North America, have found conflicting results, both a reduction [22, 23] and an increase in satisfaction when reducing the LOS [24–27].

Because no one-sided conclusion can be drawn from previous literature and there has been very little research about the satisfaction of women who have recently given birth, it is advisable to carry out further research about this topic [13, 28].

Satisfaction is a multidimensional concept influenced by a variety of factors. It is known that care providers, and how they act, have an important influence on satisfaction [20, 29]. The main care providers in the postpartum period are midwives. Both technical and emotional skills characterize the midwives and can influence the feelings of satisfaction in new mothers [11, 30]. Giving accurate breastfeeding support [11, 21, 31, 32] is one of those technical element that is important for the mothers’ satisfaction. Helpfulness, showing empathy, showing interest [21], absence of hastiness, taking anxieties and concerns seriously [33], taking time to talk through the birth experience [34], connecting with the women, involving women in the decision making process [35] are examples of emotional support in perinatal care providers. Other dimensions that have an impact on satisfaction of care are ‘Provision of information by caregivers’ [5, 21, 30–33, 36], and ‘Physical environment at the maternity ward’ [32, 34, 36] and ‘Perceived privacy’ [32, 36].

The aim of this study was to compare maternal satisfaction of postpartum care before and after the reduced LOS was implemented, by introducing the KOZI&Home care program.

## Methods

### Setting and design

The University Hospital Brussels has an average of 2400 births a year. The average LOS, prior to the intervention, was four days for a vaginal delivery, and five days following a caesarean section. The Belgian minister of health set up several pilot projects to ensure quality of care, while reducing LOS postpartum. The KOZI&Home program was set up in the University Hospital Brussels, encouraging a stay of two days or less for a vaginal birth and four days or less for a caesarean section, with extra prenatal visits and home care attached. The study design of this study was quasi-experimental, due to the incorporation of the KOZI&Home program, the KOZI&Home program is called ‘the intervention’ further in this paper. This intervention was set up to help women prepare for a shorter postpartum LOS. Additional midwifery appointments in the intervention at 16 and 36 weeks’ gestation with extra information provided about the program, home care after birth, and the role and need for having an independent midwife and general practitioner. Women were also advised to contact an independent midwife around week 28. This independent midwife visited the pregnant women at home around week 32, ensured follow-up at home in the postpartum and reported back to the hospital. Women were also given a list of pediatricians at week 36. After discharge, the following care was provided: (a) from day three to five postpartum, daily visits of the independent midwife at home; (b) between days seven and ten postpartum, a visit to the pediatrician was scheduled; (c) between weeks two to six postpartum, visits to ‘Kind en Gezin’(K&G) or to 'Office de la Naissance et de l’Enfance’(ONE) (paragovernmental organizations in charge of the follow-up for all children up to the age of six) were planned; and (d) a KOZI telephone line was also available for parents at all time for questions and concerns (see Table 1).

**Table 1:** Description of the items included in the non-intervention and intervention periods

	Non intervention	Intervention
Every two—four weeks midwife/gynecologist visit	Present	Present
Three times structured ultrasound (at 12, 20 and 32 weeks of pregnancy, 2D ultrasound [37])	Present	Present
Additional KOZI&Home visits at 16 and 36 weeks of pregnancy	Absent	Present
Independent midwife visit at home around 32 weeks of pregnancy	Possibly	Present
Independent midwife visits the first days postpartum at home	Possibly	Present
Pediatrician visit at day ten postpartum	Possibly	Present
Between weeks 2–6 postpartum K&G or ONE visits	Present	Present
Pediatrician visit at week 6 postpartum	Present	Present
Telephone help line	Absent	Present

From February 2016 to May 2016, the non-intervention study period was observed. During this period a normal care trajectory was provided as recommended in the national antenatal care guidelines [38]. In addition, women could organize extra care themselves as they wish/or needed. If they wanted an independent midwife, they could have it, but all organizing themselves. The intervention study period started in October 2017 and ended in November 2018. In the intervention group, midwife visits, an early pediatrician visit and the telephone help line were systematically organized.

### Population

Women were included at their first consultation during pregnancy if they were: > 18 years old, singleton or twin pregnancy and speaking Dutch, English, French, Turkish or Arabic. After giving birth there were some additional inclusion criteria: > 36 weeks’ gestation, having their baby in with them in their room (ie. not admitted in neonatology), birth weight > 2300 g and uncomplicated vaginal birth or planned caesarean section. In case of birth weight < p10 for gestational age, baby weight loss > 5% after 24 h and > 8% after 48 h, abnormal feeding and stool pattern, oxygen saturation < 95%, positive direct Coombs, visible icterus within the first 24 h and higher than normal bilirubin values on day two, women were not included in the intervention group. After hospital discharge, home visits with an independent midwife were scheduled, Guthrie test was performed by an independent midwife (between 72 and 96 h after birth), an extra appointment with a pediatrician was given (between days seven and ten), mother and/or father was/were coached for indications for medical advice (drowsiness, abnormal drinking, icterus, losing weight) and a standard letter for referral was provided. Herhaling, zetten bij vorige passage hierover: subtitle: subscription opf the KOZI&Home prpgram.

### Data collection

Women who agreed to participate in the study signed a consent form prior to discharge. Women were asked to complete two satisfaction questionnaires: one on the day of discharge from the maternity ward (The Maternity Satisfaction Questionnaire (MSQ), consisting of 19 questions, administered by the midwife), and one when at home, via a telephone call by the researcher, two weeks postpartum (The Home Satisfaction Questionnaire (HSQ), consisting of 13 questions). Both questionnaires were scored using a 5 point Likert scale (1, strongly disagree; 2, disagree; 3, undecided; 4, agree; 5, strongly agree), available in Dutch, English and French. For Arabic and Turkish speaking women an intercultural translator was involved. Both questionnaires were made on the basis of two already existing satisfaction questionnaires: The ‘COMFORTS’ scale [36] and the ‘SMMS-normal birth scale’ [32]. The focus was not only on satisfaction with care, but also extra questions about breastfeeding, readmissions to the hospital and support from family and friends, were asked.

The MSQ and HSQ includes several dimensions of satisfaction with care. Those dimensions are: ‘Midwives time investment’, ‘Provision of information’, ‘Physical environment’ and ‘Privacy’ [32, 36]. We added a fifth dimension ‘Readiness for discharge’, since this study mainly examines the readiness for discharge in women who just gave birth. In the dimension ‘Physical environment’, questions about the cleanliness and received rest were asked, while in the dimension ‘Privacy’ questions about the privacy caregivers and housekeeping staff gave, were asked.

Lastly, data were extracted from medical records: age, country of origin, language, marital status, education level, employment, parity, gestational age at birth, induction of labor, epidural anesthesia, type of giving birth, episiotomy, birth weight, type of room (single or shared) and LOS. This study was approved by Ethical Committee of university hospital Brussels.

### Data analysis

Baselines characteristics were described, and these characteristics were compared between both groups, using Pearson’s Chi squared tests. Satisfaction was measured using the same methodology as suggested in the ‘SMMS-normal birth scale’ and ‘COMFORTS scale’ manuals [32, 36]. Answers to each question were first analyzed by calculating means and standard deviations (SD), and thereafter summed according to the five dimensions of satisfaction with care received (‘Midwives time investment’, ‘Provision of information’, ‘Physical environment’, ‘Privacy at the maternity ward and at home’ and ‘Readiness for discharge’) and converted to a five-point Likert scale as described in the manuals. For bivariate analysis, satisfaction subscores were compared between the non-intervention and intervention group using t-tests.

In order to explore factors influencing the differences between periods, analysis was split into five parts for each of the significant subscales: (i) socio demographic background, (ii) ‘pregnancy, labour, delivery’, (iii) support women receive, (iv) readmission, and (v) breastfeeding. Regression modelling was applied on the complete data, which was a combination of forward and backward selection (both directions) based on the Akaike information criterion (AIC). The variable that indicated which program (intervention or not) the women followed were in this regression model by default. We corrected for multiple testing based using the Benjamini-Hochberg’s false discovery rate correction.

In a second phase, the significant values were added into one overall model based on the data available for those selected variables. Again, model selection in both directions was applied. After obtaining the final model, the assumptions of linearity, homoscedasticity and normality of the residuals were checked. We used the statistical package R version 4.0.03 running in RStudio 1.4.1103 for this purpose. We worked at the alpha level α = 0.05.

## Findings

### Population

**Fig. 1:**
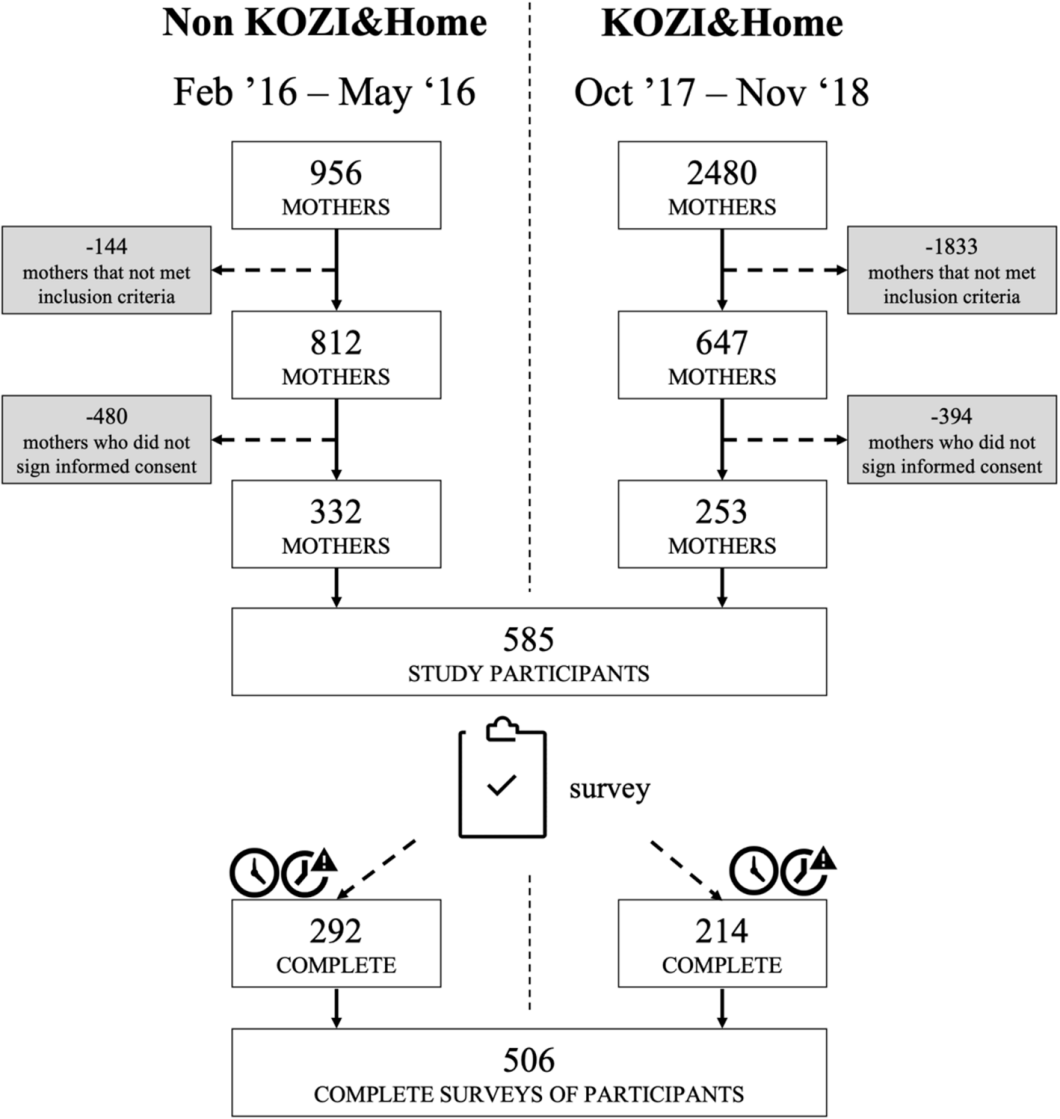
Population and sample data

During the non-intervention study-period 956 women gave birth and 2480 during the intervention study-period (see Fig. 1). Among the 956 women of the non-intervention study-period, 812 met the inclusion criteria, 332 (40.9%) of them signed the informed consent and were included, 292 women completed both surveys. From the 2480 women who gave birth during the intervention study-period, 647 women met the inclusion criteria, 253 (39.1%) agreed to participate and were included, 214 women completed both surveys.

### Women’s characteristics

Table 2 gives an overview of the population characteristics in both studied groups. Compared to the non**-**intervention group, there were more single mothers (7.2% vs 2.7%) in the intervention group (*p*=0.03). There were more multiparous women (66.5% vs 54.8%; *p*=0.005), more births between 37-40 weeks (87.3% vs 80.1%; *p*=0.01), fewer inductions of labour (23.6% vs 34.9%; *p*=0.005) and fewer episiotomies (16.0% vs 34.0%; *p*<0.001) in the intervention group compared with the non-intervention group. When comparing groups, the help from family and friends, readmission rates and breastfeeding, showed no statistically significant differences. The difference in LOS was measured and women in the intervention group had an average stay of 2.4 days, while in the non-intervention group women stayed for an average of 3.7 days.

**Table 2:** Population characteristics: comparison of both groups—Percentages are calculated on valid data

	**Non-intervention** ***N* = 332**^**a**^** (56.8%)**	**Intervention** ***N* = 253**^**b**^** (43.2%)**	**Pearson’s chi-squared test**
**Socio demographic background**	N (valid %)	N (valid %)	*P*-value
**Language**	332	252	0.39
** Dutch**	138 (41.6%)	118 (46.8%)	
** French**	181 (54.5%)	123 (48.8%)	
** Other**	13 (3.9%)	11 (4.3%)	
**Country of origin**	212	229	0.33
** Belgium**	103 (48.6%)	100 (43.7%)	
** Maghreb countries**	47 (22.2%)	47 (20.5%)	
** Other**	62 (29.2%)	82 (35.8%)	
**Age**	332	253	0.45
** ≤ 35 years**	262 (78.9%)	206 (81.4%)	
**Profession**	225	201	0.40
** Working**	140 (62.2%)	133 (66.2%)	
**Family status**	224	209	0.03
** Living together**	218 (97.3%)	194 (92.8%)	
**Education level**	202	205	0.18
** Higher education**	111 (55.0%)	126 (61.5%)	
**Income**	203	205	0.19
** Own income**	122 (60.1%)	136 (66.3%)	
**Pregnancy, labour, delivery**	N (valid %)	N (valid %)	*P*-value
**Parity**	332	245	0.005
** Primipara**	150 (45.2%)	82 (33.5%)	
** Multipara**	182 (54.8%)	163 (66.5%)	
**Childbirth**	332	220	0.11
** Vaginal birth**	280 (84.3%)	174 (79.1%)	
** Caesarean section**	52 (15.7%)	46 (20.9%)	
**Gestational age**	331	228	0.01
** 36 weeks**	13 (3.9%)	1 (0.4%)	
** 37–40 weeks**	265 (80.1%)	199 (87.3%)	
** > 40 weeks**	53 (16.0%)	28 (12.3%)	
**Birth weight**	330	250	0.40
** < 2500 g**	7 (2.1%)	2 (0.8%)	
** 2500–4000 g**	296 (89.7%)	230 (92.0%)	
** > 4000 g**	27 (8.2%)	18 (7.2%)	
**Induction of labour**	332	220	0.005
** No**	216 (65.1%)	168 (76.4%)	
**Episiotomy**	329	225	< 0.001
** No**	217 (66.0%)	189 (84.0%)	
**Epidural anaesthesia**	331	227	0.81
** Yes**	236 (71.3%)	164 (72.2%)	
**Organisation of care**	N (valid %)	N (valid %)	*P*-value
**Room type in hospital**	332	248	0.07
** Single room**	191 (57.5%)	124 (50.0%)	
** Double room**	141 (42.5%)	124 (50.0%)	
**Average LOS**	3,70 days	2,38 days	

^a^Missing’s non-intervention (*n* = 332): Country of origin *n* = 120; Profession *n* = 107; Family status *n* = 108; Education level *n* = 130; Income *n* = 129; Gestational age *n* = 1; Birth weight *n* = 2; Episiotomy *n* = 3; Epidural anaesthesia *n* = 1

^b^Missing’s intervention (*n* = 253): Language *n* = 1; Country of origin *n* = 24; Profession *n* = 52; Family status *n* = 44; Education *n* = 48; Income *n* = 48; Parity *n* = 8; Childbirth *n* = 33; Gestational age *n* = 25; Birth weight *n* = 3; Induction of labour *n* = 33; Episiotomy *n* = 28; Epidural anaesthesia *n* = 26; Room type *n* = 5

### Satisfaction in both studied groups

We found higher satisfaction scores in the intervention group compared with the non-intervention group on three of the subscales of satisfaction measured at home.

Satisfaction with ‘provision of information’ at home had a mean score of 4.47 (SD 0.73) in the intervention group versus 4.08 (SD 0.76) in the non-intervention group (*p* < 0.001). Women in the intervention group were more satisfied regarding ‘privacy at home’ (mean 4.74 (SD 0.49) versus 4.48 (SD 0.71); *p* < 0.001) in the non-intervention group. Also, women were more satisfied regarding ‘readiness for discharge’ (mean 4.20 (SD 1.00) versus mean 3.93 (SD 0.99), *p* = 0.02) in the intervention group (Table 3).

**Table 3:** Mean scores for 5 subscales of satisfaction measured at the maternity ward and at home in the non-intervention and intervention group

Satisfaction, subscales	Non-intervention Mean + SD	Intervention Mean + SD	*P*-value via T-test	Adjusted *P*-value^*^
At discharge from the MATERNITY (MSQ)				
Time of midwife	4.41 ± 0.68	4.38 ± 0.70	0.59	0.85
Provision of information	4.47 ± 0.60	4.48 ± 0.62	0.91	0.91
Evironment	4.09 ± 0.73	4.22 ± 0.71	0.03	0.08
Privacy	4.50 ± 0.57	4.46 ± 0.63	0.54	0.85
Readiness for discharge	4.58 ± 0.70	4.60 ± 0.68	0.72	0.86
At HOME (HSQ)				
Time of midwife	4.45 ± 0.77	4.42 ± 1.15	0.77	0.86
Provision of information	4.08 ± 0.76	4.57 ± 0.73	< 0.001	< 0.001
Environment	3.19 ± 0.94	3.27 ± 1.09	0.40	0.79
Privacy	4.48 ± 0.71	4.74 ± 0.49	< 0.001	< 0.001
Readiness for discharge	3.93 ± 0.99	4.20 ± 1.00	0.005	0.02

^*^Benjamini-Hochberg’s false discovery rate correction for multiple testing

### Factors that impact satisfaction subscales

Analysis from the subscales ‘Provision of information at home’ and ‘readiness for discharge’ showed no other factors, except the fact of belonging to the intervention group, that was associated with the scores.

When looking at ‘Provision of information at home’ women in the intervention group on average had a significant increase in satisfaction compared to the women in the Non-intervention. The satisfaction on average about the ‘Provision of information at home’ was higher in the intervention group (Table 4).

**Table 4:** Factors impacting higher satisfaction at home, analyses for each significant satisfaction subscale (regression modelling)

	**Model estimates** with an untransformed dependent variable (full data)				**Model estimates** where (linearity, homoscedasticity and normality) assumptions of residuals are taken care of by (1) transforming the dependent variable or by (2) removing outlying cases			
	Beta	±	SE	*P* value	Beta	±	SE	*P* value
***Subscale provision of information at home (n* = *441)***								
	Untransformed dependent variable				Transformed dependent variable (squared)			
***Intercept***	4.08	±	0.05	**-**	17.22	±	0.36	**-**
***Intervention***				** < 0.001**				** < 0.001**
- standard care	(Ref.) 0.49	±	0.07		(Ref.) 4.21	±	0.53	
- Intervention								
***Subscale privacy at home (n* = *333)***								
	Untransformed dependent variable				Removal of 2 outlying cases influencing estimates (*n* = 331)			
***Intercept***	4.73	±	0.07	**-**	4.85	±	0.06	**-**
***Intervention***				** < 0.001**				** < 0.001**
- standard care	(Ref.) 0.44	±	0.07		(Ref.) 0.42	±	0.06	
- KOZI&Home program								
***Midwife visiting home***				** < 0.001**				** < 0.001**
- No	(Ref.) -0.44	±	0.08		(Ref.) -0.53	±	0.07	
- Yes								
***Support from friends***				**0.005**				**0.005**
- No	(Ref.) 0.23	±	0.08		(Ref.) 0.20	±	0.07	
- Yes								
***Method of giving birth***				**0.014**				**0.001**
- Vaginal birth	(Ref.) -0.20	±	0.08		(Ref.) -0.23	±	0.07	
- Caesarean section								
***Subscale readiness for discharge at home (n* = *441)***								
	Untransformed dependent variable				Removal of 3 outlying cases influencing estimates (*n* = 438)			
***Intercept***	3.93	±	0.07	**-**	3.92	±	0.07	**-**
***Intervention***				**0.005**				**0.003**
- standard care	(Ref.) 0.27	±	0.10		(Ref.) 0.28	±	0.10	
- Intervention								

Home visits by the midwife, support from friends and the mode of delivery were factors that, besides belonging to the intervention group, were associated with the satisfaction measured by the subscale ‘privacy at home’. Presence of support from friends and belonging to the intervention group were positively related to this satisfaction subscale score (respectively, β = 0.20, on a score of 5 (*p* = 0.005) and β = 0.42, on a score of 5 (*p* < 0.001)) when compared to the group without support from friends or belonging to the Non- intervention program. On the other hand, giving birth by Caesarean section was associated with lower subscale scores on average (β = -0.23, on a score of 5 (*p* = 0.001)) for ‘privacy at home’, when compared to women with a vaginal birth. Furthermore, the home visits by the midwife also were associated with an average reduction in the satisfaction subscore related to measuring ‘privacy at home’ (β = -0.53, on a score of 5 (*p* < 0.001)). For the subscale ‘readiness for discharge’, the average increase associated with that level of satisfaction in the intervention group was 0.28, on a score of 5 (*p* = 0.003) compared to women belonging to the non- intervention group.

## Discussion

### Principal findings

Our findings suggest that the intervention is associated with an improvement of some components of women satisfaction with postpartum care.

Satisfaction at discharge from the maternity ward did not differ between groups. Some studies show less satisfaction in the intervention group [22, 23]. In these studies, however, LOS was reduced without providing a preparation, nor support at home.

Especially in the subcategories provision of information and privacy at the maternity ward, a negative impact could be expected, since postpartum information is provided within a shorter duration [39], and so midwives entered the room more often during this shorter stay. Nevertheless, it did not impact these satisfaction subscores (‘provision of information’ and ‘privacy’). The underlying reason for these findings might be a proper preparation of discharge since the intervention included antenatal preparation for discharge.

Regarding satisfaction with care at home, women in the intervention group, had a higher satisfaction score on three subscales (provision of information, privacy and readiness for discharge). The fact that women in the intervention were more satisfied about ‘provision of information’ could be explained by home visits made by an independent midwife allowing to provide or repeat information when needed. Our multivariable analysis did not show one element to be the key for this finding. This is in line with the available literature that showed an association between more individualised care and improved satisfaction outcome scores in general [37].

Women in the intervention group had a higher satisfaction score on the subscale ‘readiness for discharge’, measured at two weeks postpartum. Since none of the other factors taken into account in our analyses could explain this difference, the preparation provided in the KOZI&Home program might be the underlying reason. Therefore, women in the intervention group felt, probably, more prepared to go home than women with a longer LOS, since they had more support at home, so in case of questions, they could ask for help at any time and when needed.

The subscale ‘privacy at home’ was scored higher on average in the intervention group at two weeks postpartum, compared to women with a longer hospital stay (standard care). While support from family and friends seems to influence this difference in a positive way, home visits by midwives, and having a caesarean delivery, impact this relation negatively.

An equal or even higher satisfaction score when reducing LOS has been shown by other studies, especially when follow-up was organised [13, 24–27, 37]. The American College of Obstetricians and Gynaecologists strongly recommend to organise postpartum home visit(s), since they are resulting in satisfaction and health improvement for mother and child [40].

### Limitations and strengths

In Belgium, this study is the first study that measures women’s satisfaction with postpartum care at two different time points, the immediate postpartum period and two weeks after giving birth. The approach of this study is unique since not more than 2 previous studies were found [5, 41]. Belangrijker is dat satisfaction is uniek in belgie, niet epr se de questionnaires. In addition, we followed the recommendation of Britton [42], assessing both global satisfaction and satisfaction with specific dimensions as an ideal measure of satisfaction. The advantage of using dimensions is, that based on the results, changes in the organization with maternity and home care are possible in each dimension separately. Above that, in general, measuring patient satisfaction is a good indicator of healthcare quality [21]. Moreover, our results will not only serve as an evaluation of women’s satisfaction, but also as a baseline for comparison with other studies in the future.

A first limitation is the fact that, having the choice whether to participate to the intervention, only 40% of women chose to take part in the study. Because of that, we obtained a non-equivalent control group, not randomly assigned to receive, or not receive the intervention. These non-equivalent groups, implies that, although no negative effect on satisfaction scores have been found, a general implementation of the KOZI&Home program (intervention) should be monitored further on. Above that, there are some significant differences between both studied groups, which could be attributed to the discharge criteria in the intervention, or indirectly related to being ready for discharge. Another limitation was that both study programs were conducted in another time frame, so history bias could have been occurred. Next, we have used a telephone line in the intervention group, where women could call day or night with questions, which is a big strength of the study, but we do not have any information about how many times the telephone line is used. The final limitation is that there were some missing values in the results, which could have been a bias in the final findings. Taking in account all previous limitations, extra research will be required before implementing this towards other hospitals.

### Implications for practice and further studies

This study suggests that reducing LOS may improve some components of women satisfaction with postpartum care. This intervention program could be considered in other settings that aim to reduce postpartum LOS, but caution is advised, since there are some limitations, mentioned above.

Future research protocols could compare different types of integrated home /follow-up care trajectories. Heath care systems could invest in the best possible postpartum care, achieving high satisfaction scores. Besides, research is needed to determine whether shortening LOS postpartum is safe in the context of readmissions/morbidity/feeding issues and that it entails a cost reduction. So, the economic impact should be considered since this is not done in this study. But some literature can already demonstrate that such care programs are cost-effective, including similar programs of postpartum care [43–45].

## Conclusion

We can conclude that there is no negative effect on reducing LOS on women’s satisfaction with care received at the maternity ward and at home, following the intervention. Even a higher satisfaction score at home was observed in the subcategories provision of information and readiness for discharge. Our study concludes that this integrated care program is acceptable for postpartum women and associated with some favourable outcomes.

## Data Availability

The datasets used and/or analysed during the current study are available from the corresponding author on reasonable request.
